# Updates on the role of miR-193b in the pathogenesis of human cancers and other diseases

**DOI:** 10.1016/j.bbrep.2025.102392

**Published:** 2025-12-02

**Authors:** Sheyda Khalilian, Mohadeseh Fathi, Sara Arezi, Soudeh Ghafouri-Fard

**Affiliations:** a Student Research Committee, School of Medicine, Shahid Beheshti University of Medical Sciences, Tehran, Iran; b Department of Medical Genetics, Shahid Beheshti University of Medical Sciences, Tehran, Iran; c Department of Hematology and Blood Banking, School of Allied Medical Sciences, Shahid Beheshti University of Medical Sciences, Tehran, Iran

**Keywords:** Non-coding RNA, miRNA, miR-193b, Cancer

## Abstract

MicroRNAs (miRNAs) have emerged as pivotal regulators of gene expression, fundamentally influencing cellular processes such as proliferation, differentiation, and apoptosis. Among these, miR-193b has garnered significant attention as a potent tumor suppressor in some contexts, while functioning as an oncogene in other situations. Its frequent loss in some types of cancers such as gastric and cervical cancers as well as osteosarcoma positions it not only as a valuable prognostic indicator but also as a potential tool for gene therapy. On the other hand, it has been found to be up-regulated in esophageal and bladder cancers. Meanwhile, data regarding its expression pattern in some types of cancers, such as colorectal, lung and pancreatic cancers is conflicting. Moreover, its influence extends beyond oncology, implicating it in a diverse range of human diseases, including systemic sclerosis, aortic dissection, osteoarthritis, diabetes, psoriasis, Parkinson's disease, Alzheimer's disease, sepsis and allergic rhinitis. However, miR-193b does not have a single, unified function; instead, its role is highly context-dependent, acting as either a protective molecule or a pathogenic driver depending on the specific tissue and disease. This review aims to consolidate the current updates on the role of miR-193b, detailing its molecular mechanisms, validated target genes, and its burgeoning potential as a diagnostic biomarker and therapeutic agent in cancer and other pathological conditions.

## Highlights

• miR-193b is a potent tumor suppressor in some contexts, and an oncogene in other situations.
• It is implicated in a diverse range of human diseases.
• It is a diagnostic biomarker and therapeutic agent in cancer and other pathological conditions.

## 1 Introduction

The post-genomic era has uncovered the great significance of non-coding RNAs, with microRNAs (miRNAs) standing out as master regulators of post-transcriptional gene silencing. Dysregulation of these small RNA molecules is a hallmark of numerous pathological states, particularly cancer. Various studies have evaluated miRNA expression patterns and functions across different cancer types [[1], [2], [3]]. The great interest in this field is mainly due to the miRNA stability in circulating or other biofluids, and their potential as biomarkers for diagnostic and follow-up purposes. hsa-miR-193b (MI0003137) has been consistently identified as a key player in the carcinogenesis with both tumor-suppressive and oncogenic functions. This miRNA is encoded by *MIR193B* gene located in chr16:14,303,967–14,304,049 (GRCh38/hg38) Plus strand (Size: 83 bases). Its downregulation, mediated by promoter hypermethylation [4] or other mechanisms, leads to the unchecked expression of a number of oncogenes like CCND1 [5], ETS1 [6], and PLAU [7], thereby driving tumorigenesis, metastasis, and chemoresistance. Meanwhile, it exerts oncogenic roles in some types of cancer through down-regulating a number of tumor suppressors. Furthermore, emerging evidence positions miR-193b at the crossroads of other human diseases, including metabolic, inflammatory, and neurodegenerative disorders. This review provides a comprehensive overview of the latest research, synthesizing our current understanding of miR-193b′s multifaceted roles, its utility in clinical settings, and the challenges and opportunities in harnessing its power for future therapeutics. Additionally, the biological and molecular mechanisms of miR-193b functions as well as its regulatory mechanisms are discussed in the physiological and the pathological context. Finally, we highlight dysregulation of miR-193b in affected tissues and blood samples in several pathological conditions, particularly cancer.

## 2 Search strategy

In the current narrative review, our objective was to identify and synthesize recent literature on the role of miR-193b (2019–2025) in any human disease, with a particular focus on cancer. We searched PubMed/MEDLINE and Google Scholar with the search string being structured in three core blocks: Block A: miR-193b; Block B: The Functional Role ((“Gene Expression Regulation” OR “Carcinogenesis” OR “Tumor Suppressor” OR “Oncogene” OR “OncomiR” OR “Pathogenesis” OR “Pathway” OR “Biomarker” OR “Diagnostic” OR “Prognostic” OR “Therapeutic” OR “Target” OR “Proliferation” OR “Apoptosis” OR “Migration” OR “Invasion” OR “Metastasis”); and Block C: Disease Context (Neoplasms OR Cancer OR Tumor OR Carcinoma OR Oncology OR Leukemia OR Lymphoma OR Non-malignant disorders). The final search combined these blocks as: **A AND (B OR C).** The search was restricted to English language. For article type, we initially had no restriction, but review articles were flagged for manual screening of their reference lists. All retrieved records were then exported into EndNote and duplicate records were removed. Subsequently, two independent reviewers screened titles and abstracts against pre-defined inclusion/exclusion criteria. Original research, case reports, or reviews that investigated miR-193b in the context of any human disease or biological process were included. Studies not involving humans or human-derived cell lines/tissues and studies not focused on miR-193b were excluded. Finally, data from included studies was extracted and tabulated capturing: Cancer/Disease Type, Expression Pattern, Samples/Cell Lines, Methods, Pathways/Interactions, Function, and References.

## 3 Role of miR-193b in cancer

Table 1 comprehensively summarizes the current research findings on the role of miR-193b across a wide spectrum of human cancers. In this table, data is organized into key categories, including the type of cancer, observed expression pattern of miR-193b, the samples and cell lines used, the experimental methods employed, the implicated molecular pathways or direct interactions, the proposed biological function of miR-193b, and the corresponding reference.

**Table 1:** Summary of cell line/human studies on the role of miR-193b in cancers.

Type of cancer	Expression Pattern	Samples	Cell lines	Methods	Pathway/interaction	Function	Reference
Lung cancer	Down	Human lung adenocarcinoma cell lines	CL1-1, CL1-5	Immunohistochemistry, Quantitative real-time PCR, Luciferase assay, Western blotting ChIP assay	PRNP mRNA	miR-193b-3p acts as a tumor suppressor via modulating the c-Jun/miR-193b-3p/PRNP signaling pathway.	(8)
Lung adenocarcinoma	Down	–	A549 cell line	Western blot assay Microarray Quantitative Real-Time PCR Assay	–	miR-193b-3p acts as a tumor suppressor in acidic conditions.	(9)
Lung adenocarcinoma	Down	Tissue samples from 11 patients with lung adenocarcinoma	–	Quantitative Real-Time PCR Assay	–	miR-193b-3p acts as tumor suppressor through interaction with apoptosis pathway.	(10)
Lung adenocarcinoma	Down	–	A549 cell lines	Quantitative Real-Time PCR Assay	–	miR-193b shows potential as a diagnostic biomarker for LUAC	(11)
Non-small cell lung cancer	Up	serum samples from 101 patients with NSCLC and 28 controls	–	Quantitative Real-Time PCR Assay	–	miR-193b demonstrates a notable correlation with metastasis.	(12)
Non-small cell lung cancer	Up	32 pairs of NSCLC tissue samples and adjacent control tissue specimens	–	Quantitative Real-Time PCR Assay	–	Abnormal expression of miR-193b-3p could potentially act as a biomarker for non-small cell lung cancer (NSCLC).	(13)
Non-small cell lung cancer	Up	Serum and tissue samples from 185 patients with NSCLC	–	Quantitative Real-Time PCR Assay	Wnt signaling pathways, insulin signaling pathways, apelin signaling pathways	miR-193b-3p acts as oncomiR in NSCLC, however, its specific role remains unclear.	(14)
Esophageal squamous cell carcinoma	Up	*In silico* dataset analysis	–	Bioinformatics analysis	–	miR-193b-5p shows ability to predict response to neoadjuvant therapy.	(15)
Esophageal Cancer	Down	Human esophageal cancer cell lines	C9706, KYSE30, KYSE70, KYSE150, KYSE180, KYSE410, KYSE450, KYSE510	Immunohistochemistry Assay, Quantitative Real-Time PCR Assay, Flow cytometry, MicroRNA Immunoprecipitation (RIP) Assay	RSF1 (Remodeling and Spacing Factor 1)	miR-193b-3p acts as a tumor suppressor in ESCC by interacting with RSF1.	(16)
Pancreatic ductal adenocarcinoma	Down	FFPE specimens from 179 PDAC patients and 171 normal pancreatic tissues	–	Quantitative Real-Time PCR Assay	miR-193b/KRAS/LAMC2, XPO1/KRAS	miR-193b acts as a sponge for LAMC2 and KRAS, leading to the inhibition of the AKT/ERK downstream pathway in PDAC.	(17)
Pancreatic cancer	Up	–	SW1990 cells	Luminescence assays Quantitative Real-Time PCR Assay	TRIM62	miR-193b-3p acts as oncomiR by enhancing the proliferation, migration, invasion, and glutamine uptake of SW1990 cells.	(18)
Pancreatic Cancer	Down	–	PDAC cell lines	Quantitative Real-Time PCR Assay	eEF2K	miR-193b-3p acts as tumor suppressor by obstructing the eEF2K/MAPK-ERK oncogenic pathway in PDAC.	(19)
Colorectal cancer	Up	*In silico* dataset analysis	–	Bioinformatic tools	–	Expression of miR-193b-3p is elevated in patients at stages I and II, but it diminishes as the disease advances.	(20)
Colorectal cancer	Up	–	HCT116, HEK293T, HT29, SW480, SW620, HCT15, LIM2527	Quantitative Real-Time PCR Assay	TRF2	miR-193b-3p acts as oncomiR in CRC through interaction with TRF2/CTCF	(21)
Colorectal cancer	Up	*In silico* dataset analysis	–	Bioinformatic analysis	–	miR-193b-3p acts as a noninvasive biomarker in patients with colorectal cancer	(22)
Colorectal cancer	Down	–	HEK-293 T	Quantitative Real-Time PCR Assay	LncRNA CRART16, HMGA2	miR-193b-5p acts as a tumor suppressor. CRART16 inhibits expression of miR193b-5p through resistance to 5-FU.	(23)
Colon cancer	–	*In silico* dataset analysis	–	Bioinformatic analysis	Hub genes (TOP2A, PCNA, RTKN, MCM7, SHMT2, CDCA5, CDCA7,..)	miR-193b-3p acts as a suppressor and inhibits metastatic activity of colon cancer cells.	(24)
Head and neck squamous cell carcinoma	–	12 HNSCC tumor samples	CAL27	scRNA sequencing data processing, Quantitative Real-Time PCR Assay	JPX/miR-193b-3p/PLAU	Highest activity of the JPX/miR-193b-3p/PLAU axis in malignant epithelial cells results in increased cell proliferation, migration, and invasion in HNSCC.	(7)
Head and neck squamous cell carcinoma	–	Tissue samples from 5 patients with HNCC and 5 controls	–	Microarray analysis	tryptophan 5-monooxygenase activating protein ZETA (14-3-3ζ)	miR-193b-5p acts as a oncomiR via modulates the expression of tryptophan 5-monooxygenase activator protein ZETA	(25)
Tongue squamous cell carcinoma (TSCC)	–	15 pairs of TSCC tissue and adjacent control specimens	–	Flow cytometry, Western Blot, Quantitative Real-Time PCR Assay	PI3K/AKT signaling pathway	LINC00152 sponges miR-193b-3p, facilitating the phosphorylation and activation of the PI3K pathway, thereby playing a role in the development of TSCC.	(26)
Tongue cancer	Down	–	TCA-8113, CAL27	Fluorescence quantitative PCR (qPCR), Western blotting, Flow cytometry analysis	AKT/mTOR signaling pathway	miR-193b-3p acts as a tumor suppressor via inhibiting the AKT/mTOR pathway. It mimics apoptosis in CAL27 and TCA-8113 cells by downregulating Bcl2 expression and enhancing the levels of Active-Caspase3 and Bax.	(27)
Nasopharyngeal cancer	Up	17 metastatic and 17 non-metastatic NPC tissues and Peripheral blood samples were collected from 21 patients with NPC	CNE-2	Quantitative Real-Time PCR Assay Flow cytometry	mitogen-activated protein*/*MEKK3	miR-193b acts as a oncimiR via facilitates the activation of TAM by directly influencing MEKK3.	(28)
Cutaneous Melanoma	Down	cutaneous melanoma plasma samples and normal plasma samples (control)	–	Quantitative Real-Time PCR Assay	*-*	miR-193b-3p acts as a noninvasive biomarker.	(29)
Melanoma	Down	–	A‐375, SKMel-13, SK‐Mel‐19, SK-Mel‐23, Mel-HO, JPC‐298, Mel‐2a, Mel-JuSo, MeWo	Western Blot, Quantitative Real-Time PCR Assay	TRAIL	miR-193b acts as a tumor suppressor via induced expression of TRAIL in melanoma.	(30)
Breast cancer	Up	80 patients diagnosed with ABC were treated using CDK4/6 inhibitors.	–	Quantitative Real-Time PCR Assay	*-*	miR-193b is capable of distinguishing between patients with responders and non-responder to CDK4/6 inhibitor therapy.	(31)
Breast Cancer	Down	Serum sample from 58 patients with BC and 36 patients with benign tumor	–	Western Blot Quantitative Real-Time PCR Assay	CD44v6	miR-193b-3p acts as a tumor suppressor via inhibits CD44v6 expression.	(32)
Triple negative breast cancer	Down	–	MDA-MB-231 TNBC	microRNA gain-of-function experiments	WNT/β-catenin, c-Met signalling pathways	miR-193b acts as tumor suppressive through interaction with WNT/β-catenin, c-Met signalling pathways	(33)
Human Meningiomas	Up	FFPE Specimens from meningioma samples	–	Western Blot Quantitative Real-Time PCR Assay	CCND1 3′UTR	miR-193b-3p modulates the proliferation of meningioma cells by negatively regulating the expression of cyclin D1.	(34)
Ovarian cancer	–	*In silico* dataset analysis	–	Microarray data and gene expression profile analysis	CENPU, MELK, CDCA5, CDCA7, MCM10, KIF11, TRIP13, ECT2, KIF15	key miRNA that targets HGPT genes.	(35)
Endometrial Cancer and Ovarian Cancer	–	*In silico* dataset analysis	–	Bioinformatic analysis	–	miR-193b-3p modulates the expression levels of Differentially Expressed Genes (DEGs)	(36)
Cervical cancer	Down	–	HEK293	Western Blot Quantitative Real-Time PCR Assay	PI3K-AKT	Downregulation of miR-193b-3p serves a functional role in the regulation of the PI3K-AKT pathway.	(37)
Cervical Cancer	Down	41 pairs of cervical cancer tissue and adjacent control tissue specimens	–	Western Blot Quantitative Real-Time PCR Assay	CCND1	miR-193b acts as tumor suppressor, and its downregulation increases the aggressiveness of cervical cancer by targeting CCND1.	(5)
Hepatocellular carcinoma	–	*In silico* dataset analysis	–	Bioinformatics analysis	*-*	miR-193b-3p can function as an essential upstream regulatory factor for UBE2C in HCC.	(38)
Hepatocellular Carcinoma	Down	Two cohorts of patients with HCC (cohort1:n = 36 Cohort2:n = 81)	–	miRNA Profiling and Bioinformatics Analysis Quantitative Real-Time PCR Assay	*-*	Sorafenib led to an increased accumulation of miR-193b-3p within exosomes. Consequently, the influence of Sorafenib on miRNA expression demonstrated an anti-tumoral effect by diminishing migration in HepG2 cells.	(39)
Hepatocellular carcinoma patients	Down	Tissue samples from 36 patients with HCC	–	Quantitative Real-Time PCR Assay	–	No notable correlation was found between miR-193b and various clinicopathological characteristics of the patients.	(40)
Hepatocellular carcinoma	Up	–	WRL-68 THLE-3, HepG2, Huh7, MHCC97, HCCLM	Western Blot Quantitative Real-Time PCR Assay	Interaction with GASAL1	GASAL1 sponges miR-193b-5p to promote HCC cell proliferation	(41)
Hepatocellular carcinoma	Down	10 pairs of HCC tissue samples and adjacent control tissue specimens	–	Flow cytometry, Western Blot, Quantitative Real-Time PCR Assay	LncRNA H19	miR-193b-3p functions as a tumor suppressor. The lncRNA H19 sequesters miR-193b-3p. lncRNA H19 exhibits a negative correlation with miR-193b while showing a positive correlation with the MAPK1 gene.	(42)
Hepatoblastoma	Up	*In silico* dataset analysis	–	Bioinformatics analysis	–	Valuable therapeutic targets or prognostic markers for forthcoming research on Hepatoblastoma	(43)
Seminoma	Up	–	Tcam 2	–	ZBTB7A	Exosomal miR-193b-3p modulates the chemosensitivity of TCam-2 cells to cisplatin via ZBTB7A signaling.	(44)
Bladder cancer	Up	Tissue samples from 99 patients with BC and 125 controls	–	Quantitative Real-Time PCR Assay	–	miR-193b-3p acts as an oncomiR in BC.	(45)
Acute myeloid leukemia	Up	228 patients diagnosed with AML	–	Quantitative Real-Time PCR Assay	NOTCH1	miR-193b-3p acts as oncomiR in AML by upregulating NOTCH1.	(46)
Acute myeloid leukemia	Down	Bone marrow samples from 57 patients with AML	–	Flow cytometry, Western Blot, Quantitative Real-Time PCR Assay	LncRNA SNHG14 MCL1	miR-193b-3p acts as a tumor suppressor via targeted MCL1. LncRNASNHG14 silencing resulted in reduced viability and increased apoptosis in AML cells through the modulation of the miR-193b-3p/MCL1 axis.	(47)
B-chronic lymphocytic leukemia (B-CLL)	–	140 CLL patients with treatment-naive (18–65 years)	–	Quantitative Real-Time PCR Assay	–	Presence of miR-193b is associated with a particular probability of response rate to the FCR treatment outcome.	(48)
T-cell acute lymphoblastic leukemia	Down	Bone marrow samples from 42 patients and 19 controls	–	Quantitative Real-Time PCR Assay	NOTCH1	miR-193b-3p acts as a tumor suppressor. sponging miR-193b-3p by circ_0000745	(49)
Diffuse large B cell lymphoma	Up	156 patients received treatment with R–CHOP	–	Quantitative Real-Time PCR	–	miR-193b-5p up-regulation are associated with the lack of efficacy of standard chemotherapy in diffuse large B-cell lymphoma (DLBCL).	(50)
Gastric cancer	Down	–	AGS, MGC-803	Quantitative Real-Time PCR	METTL3	miR-193b-5p acts as a tumor suppressor and is inhibited by BLACAT2	(51)
Osteosarcoma	Down	–	F4, F5M2, 293A	Western Blot Quantitative Real-Time PCR Assay Chromatin immunoprecipitation (ChIP)-qPCR	MYC	miR-193b interacts with MYC, inhibiting the growth and metastasis of osteosarcoma.	(52)

Collectively, miR-193b has dual role as both tumor suppressor and oncomiR. It frequently acts as a tumor suppressor, with its expression being downregulated in numerous cancers such as lung adenocarcinoma, esophageal cancer, triple-negative breast cancer and cervical cancer. In these contexts, its loss is associated with increased cell proliferation, migration, invasion, and chemotherapy resistance. Conversely, in other cancers like non-small cell lung cancer (as a whole group; specifically in serum and tissue studies), nasopharyngeal cancer and bladder cancer, miR-193b is reported to be upregulated and functions as an oncomiR, promoting tumor growth and progression. In some other types of cancer, such as hepatocellular carcinoma, pancreatic cancer, colorectal cancer and acute myeloid leukemia, no consistent expression pattern is found.

Mechanistically, miR-193b exerts its effects by targeting crucial genes and signaling pathways involved in oncogenesis. Commonly implicated pathways include: proliferation and cell cycle (via targets like CCND1 (Cyclin D1) [34] and MYC [52]), survival and apoptosis (through regulators such as MCL1 [47] and the PI3K/AKT/mTOR pathway [26]), invasion and metastasis (through interacting with PLAU (uPA) [7], KRAS/LAMC2 [17], and Wnt/β-catenin signaling [14]. More importantly, miR-193b can modulate sensitivity to drugs like cisplatin [44] and 5-FU [23].

miR-193b′s activity is often fine-tuned by competing endogenous RNA (ceRNA) networks. Several long non-coding RNAs (lncRNAs), such as H19 [42], LINC00152 [26], CRART16 [23], and SNHG14 [47], are shown to “sponge” or sequester miR-193b, thereby releasing its mRNA targets from its inhibitory roles.

A significant number of studies position miR-193b as a promising diagnostic, prognostic, or predictive biomarker. Its expression levels, detectable in tissues, serum, and plasma, are correlated with disease stage [20], metastasis [12], and response to therapies like CDK4/6 inhibitors in breast cancer [31] and R–CHOP in lymphoma [50]. This underscores its potential utility in non-invasive liquid biopsies and personalized medicine.

Taken together, miR-193b is a multifaceted miRNA whose role is highly specific to the cancer type and biological context. Its dual function as a tumor suppressor or oncomiR, its regulation by complex networks, and its strong biomarker potential are the key features that deserver additional studies.

## 4 Role of miR-193b in lung cancer

Role of miR-193 has been vastly assessed in the context of lung cancer. For instance, Ho et al. have shown a tumor suppressor role for miR-193b-3p in this type of cancer that is mainly exerted through PRNP targeting [8]. Overexpression of this miRNA suppresses lung cancer cell migration, invasion and proliferation through this axis. Most notably, high PRNP or low miR-193b-3p levels have been correlated with poor overall survival. Additional studies have shown the role of c-Jun as a transcriptional repressor of miR-193b-3p, emphasizing on the role of c-Jun/miR-193b-3p/PRNP axis as a new therapeutic target for combating lung cancer metastasis [8]. Additionally, miR-193b-3p expression has been found to be reduced under acidic *in vitro* conditions. Downregulation of this miRNA has been accompanied with up-regulation of TGFβ2, leading to epithelial-mesenchymal transition (EMT) in A549 cells. Therefore, the interplay between miR-193b-3p, TGFβ2, and the acidic microenvironment within tumors is a possible mechanism for EMT changes [9]. On the other hand, three other studies have demonstrated an oncogenic role for miR-193b in non-small cell lung cancer (NSCLC). First, miR-193b has demonstrated a notable correlation with metastasis in a certain cohort of patients [12]. Moreover, over-expression of miR-193b-3p could potentially act as a biomarker for NSCLC [13]. Finally, another study has reported that miR-193b-3p acts as an oncomiR in NSCLC, however, its specific role remains unclear [14].

## 5 Role of miR-193b in colorectal cancer

The majority of studies on the role of miR-193b in the colorectal cancer (CRC) point to an oncogenic role for this miRNA. It has been among three miRNAs that can effectively identify lymph node metastasis-positive T1 CRC patients who received upfront surgery [20]. Besides, miR-193b-3p acts as an oncomiRNA in CRC and it is positively correlated with TRF2 expression. In fact, its expression is controlled by a cooperative activity of TRF2 and the chromatin organization factor CTCF. Additional studies in the CRC patients have verified the translational relevance of the oncogenic properties of this miRNA and revealed the prognostic value of association between TRF2 and miR-193b-3p [21].

## 6 Role of miR-193b in pancreatic cancer

Data regarding the function of miR-193b in pancreatic cancer is conflicting. Kirtonia et al. have identified a correlation among miR193b/KRAS/LAMC2, XPO1/KRAS, and LAMC2/KRAS in the pancreatic cancer cells [17]. Moreover, they have demonstrated the effect of eltanexor treatment on the expression of miR-193b which suppresses the AKT/ERK signaling in these cells [17]. This data points to a tumor suppressor role of this miRNA. However, another study has shown that miR-193b-3p content of M2 macrophage exosomes promotes the glutamine uptake, proliferation, and other malignant characteristics of pancreatic cancer cells [18]. These effects are mediated through inhibition of TRIM62, a tumor suppressor that promotes c-Myc ubiquitination. Notably, over-expression of miR-193b-3p and c-Myc has been found to be predictor of poor prognosis, while TRIM62 has an opposite effect [18].

## 7 Role of miR-193b in non-malignant disorders

miR-193b has multifaceted roles in the pathogenesis of a wide spectrum of non-cancerous diseases. In fact, miR-193b does not have a single, unified function; rather, its role is highly context-dependent, acting as either a protective molecule or a pathogenic driver depending on the specific tissue and disease.

As shown in Table 2, human studies and cell line experiments show that miR-193b is a critical regulatory molecule involved in vascular diseases, metabolic disorders, inflammatory conditions, and neurodegeneration. Its expression is frequently dysregulated (either increased or decreased) in diseased tissues compared to healthy controls, and it exerts its effects by targeting key genes in pathways controlling cell proliferation, inflammation, metabolism, and matrix remodeling.

**Table 2:** Summary of cell line/human studies on the role of miR-193b in non-malignant disorders.

Type of disorder	Expression Pattern	Samples	Cell lines	Pathway/Downstream targets	Function	Reference
Systemic Sclerosis (SSc)	Downregulated	Skin biopsies and fibroblasts from SSc patients (n = 33; 5 diffuse, 28 limited) and healthy donors (n = 25)	Human Pulmonary Artery Smooth Muscle Cells (HPASMCs)	↓ miR-193b → ↑ uPA → ↑ HPASMC Proliferation & ↓ Apoptosis → Proliferative Vasculopathy	Promotes vascular smooth-muscle proliferation and survival, contributing to proliferative vasculopathy and intimal hyperplasia in SSc	(53)
Aortic Dissection (AD)	Downregulated	Thoracic aortic tissue from AD patients (n = 25) vs. normal aortic tissue from valve-replacement controls (n = 15)	Human Aortic Smooth Muscle Cells (HASMCs)	LncRNA H19 → ↓ miR-193b-3p → ↑ MMP-2, MMP-9 & ↓ α-SMA, SM22α → ↑ Proliferation & Migration of HASMCs	Maintains vascular smooth-muscle contractility and aortic wall stability by repressing MMPs and supporting contractile proteins	(54)
Osteoarthritis (OA)/Cartilage Aging	Upregulated with age	Cartilaginous tissue from OA (n = 17), ACL injury (n = 3), and polydactylism (n = 6)	Primary human chondrocytes	↑ miR-193b in aged/dedifferentiated chondrocytes Regulates COL2A1, aggrecan and SOX9 expression; may also target SOX5 Regulates ETV1 (ER81) and E2F6 ETV1 gives positive feedback on MMP1, MMP3 (stromelysin-1) and MMP13. E2F6 is involved in feedback on ADAMTS5 and TGF-β1.	Promotes extracellular-matrix degradation, reduces matrix synthesis and may drive chondrocyte senescence/cartilage degeneration	(55)
Influenza A Virus (IAV) Infection	Downregulated	Not specified (cellular model)	A549, HEK 293, HEK293T, Madin-Darby Canine Kidney (MDCK) epithelial cells	miR-193b → ↓ β-catenin (CTNNB1) & LEF1 (Wnt/β-catenin pathway) → ↓ cyclin D1 → G0/G1 arrest → delayed vRNP nuclear import & suppressed IAV replication.	Suppresses influenza A virus replication via inhibition of the Wnt/β-catenin pathway	(56)
Type 2 Diabetes (T2D)	Upregulated	Human plasma (113 cases, 113 controls)	HepG2	↑ miR-193b → ↓YWHAZ & ↓SOS2 ↓ YWHAZ → ↑ FOXO1 → ↑ PCK1 → ↑ Hepatic Glucose Production (Gluconeogenesis) ↓ SOS2 → ↑ Insulin Resistance → ↓ IR signaling & ↓ GLUT2 → ↓ Glucose Uptake	Impairs glucose metabolism by enhancing hepatic gluconeogenesis and reducing glucose uptake; associated with increased diabetes risk.	(57)
Type 2 Diabetes (T2D) & Sarcopenia (Muscle Atrophy)	Upregulated in serum and skeletal muscle	Human Serum from 20 healthy controls (n = 20) vs. T2D (n = 20)	C2C12 mouse myoblast	↑miR-193b → PDK1↓ → Akt/mTOR/S6K pathway inactivation → ↓Protein Synthesis & FOXO1 activation → ↑Atrogin-1 and MuRF1 → ↑Protein Degradation	Impairs muscle growth by inhibiting protein synthesis and promoting degradation, leading to atrophy; inhibition attenuates muscle loss	(58)
Prediabetes/Impaired Glucose Tolerance (IGT)	Upregulated	Serum from 92 men: controls (n = 29), IFG (n = 22), IGT (n = 21), T2D (n = 20); validated in 2nd cohort: controls (n = 12), prediabetics (n = 6)	Not investigated	Not investigated	Proposed diagnostic biomarker for prediabetic state; levels correlate with post-challenge glucose, triglycerides, and fatty liver index and Levels decrease upon therapeutic intervention	(59)
Psoriasis	Downregulated	Skin tissue (patients n = 6, controls n = 5)	HaCaT, NHK	↓ miR-193b → ↑ ERBB4 protein → Activation of STAT3 & NF-κB pathways → Keratinocyte Hyperproliferation & Inflammatory Secretion	Downregulation promotes keratinocyte proliferation and inflammatory-factor secretion	(60)
Parkinson's Disease (PD)	Upregulated in PD patient PBMCs and chronic MPP + model; downregulated in acute MPP + model	PBMCs from PD patients (n = 20) and age-matched controls (n = 20)	SH-SY5Y neuroblastoma cells	↑ miR-193b → ↓ PGC-1α → ↓ FNDC5/BDNF & ↓ TFAM → ↓ Mitochondrial Biogenesis, ↑ Oxidative Stress, ↓ Neurotrophic Support → Neuronal Dysfunction and Apoptosis	Inhibits neuroprotective pathways and promotes PD progression	(61)
Parkinson's Disease (PD)	Downregulated in early PD; upregulated in late PD	Human Brain Tissue (Frozen: Controls n = 25, Patients n = 53; FFPE: Controls n = 14, Patients n = 40) Human Cerebrospinal Fluid (Controls n = 22 Patients n = 41)	SH-SY5Y neuroblastoma cells	Late PD: ↑ miR-193b→ ↓ PGC-1α →↓ Mitochondrial biogenesis & antioxidant/anti-inflammatory responses (NFE2L2, SOD2, NOS1/2, IL1B) → Cellular stress, neuroinflammation & neuronal insulin signaling	Key negative regulator of PGC-1α; upregulation in late PD impairs mitochondrial function and disrupts brain insulin signaling, linking metabolic and inflammatory dysfunction to neurodegeneration	(62)
Sepsis & Septic Shock	Downregulated	Discovery Phase: patients with sepsis (n = 6), septic shock (n = 6), controls (n = 3) Validation Phase: patients with sepsis (n = 30), septic shock (n = 30), controls (n = 30)	Not investigated	↓ miR-193b → loss of repression → ↑ NFATC2, MAP2K7, CLDN2 → Hyperactivation of TCR signaling → Excessive inflammation	Acts as an anti-inflammatory brake; its downregulation leads to immune hyperactivation and correlates inversely with CRP, PCT, IL-6	(63)
Allergic Rhinitis (AR)	Downregulated	Nasal mucosa from AR patients and healthy volunteers	Human Nasal Epithelial Cells (HNECs)	↑miR-193b → ↓ETS1 → ↓TLR4 → ↓ GM-CSF, eotaxin, MUC5AC	Represses IL-13-induced inflammatory responses in nasal epithelial cells by suppressing ETS1/TLR4 signaling	(6)
Placenta Accreta Spectrum (PAS)	Upregulated	Human term placenta tissues (n = 8 for histological analysis/miRNA localization)	JEG-3 (main functional model) BeWo (syncytialization model) Jurkat T cells (immune cell model for EV studies) HTR-8/SVneo (used as reference)	Targets K-Ras, AKT1, TMPPE, SHMT2, PTK2, MCL1(validated) and NF1, ARPC5 (potential)	Regulates key trophoblast functions by inhibiting migration, syncytialization, and apoptosis while promoting proliferation. Mediates immunosuppressive effects, reducing T-cell proliferation through extracellular vesicles	(64)
In-Stent Restenosis (ISR)	Downregulated	PBMCs from 63 subjects: ISR (n = 21), stent non-restenosis (n = 21), controls (n = 21)	Not used (direct PBMCs analysis)	Direct Target: PLAU (Plasminogen Activator, Urokinase) gene.	Downregulation leads to elevated PLAU, promoting neointimal hyperplasia and ISR pathogenesis	(65)
Multiple Sclerosis (MS)	Downregulated	Blood samples from 30 MS patients (n = 30) and controls (n = 30)	Not specified beyond luciferase assay	Unknown	Likely acts as a repressor of MS-related genes; Its downregulation/sponging by circ-cnot11-0001 is proposed to lead to the dysregulation of gene expression that contributes to MS pathogenesis	(66)
Myocardial Hypertrophy	Not directly stated for miR-193b-5p	Not specified	Not specified	LncRNA N29 → miR-193b-5p/TGFBR2 axis → Smad2/3 regulation	Is mediated by lncRNA N29 to regulate the TGFBR2/smad2/3 axis and mitigate the progression of myocardial hypertrophy	(67)
Pulmonary Vascular Dysfunction associated with Diabetes/Metabolic Syndrome (HFpEF/EIPH/CpcPH context)	Upregulated	Primary human Pulmonary Artery Vascular Smooth Muscle Cells (PAVSMCs) from diabetic (n = 6) and non-diabetic controls (n = 6).	Rat and human PAVSMCs	Metabolic Stress → ↑ROS → H3K9 Acetylation of miR-193b Promoter → ↑ miR-193b → NFYA mRNA Degradation → ↓ NFYA → ↓ sGCβ1 Transcription→ ↓NO-sGC-cGMP Signaling → Impaired Pulmonary Arterial Vasodilation	Links metabolic stress/ROS to pulmonary arterial dysfunction by degrading NFYA and shutting down the NFYA-sGCβ1-cGMP vasodilatory pathway	(68)
Endometriosis	Downregulated in eutopic endometrium of women with endometriosis; upregulated in ectopic lesions (endometrioma)	Cohort 1: 32 Women (endometriosis n = 15, controls n = 17) Cohort 2: 10 Women with endometrioma (paired ectopic & eutopic tissues collected)	Human immortalized epithelial endometriotic cell (12Z), Endometrial stromal cell (HESC)	↑miR-193b → ↓ DKK1, MIEN1, GRB7, PIK3R1, KRT19 (direct/indirect targets) → ↓ activity of migration-related pathways (Wnt, PI3K–Akt, focal adhesion, cell adhesion) → Suppression of endometrial cell migration *in vitro* → May potentially limit spread of endometriotic lesions in vivo	Suppresses endometrial cell migration; loss in eutopic tissue may promote implantation, while upregulation in ectopic lesions may limit spread	(69)
Hypertensive Disorder Complicating Pregnancy (HDCP)	Upregulated (increases with disease severity)	120 HDCP patients vs. 120 healthy pregnant women and 120 non-pregnant controls	Not investigated	Works synergistically with Neurogulin-1 (NRG-1). NRG-1 may suppress Endocrine Gland-derived Vascular Endothelial Growth Factor (EG-VEGF) expression and block ERK signaling.	Associated with onset, progression, and prognosis of HDCP; potential diagnostic and prognostic biomarker	(70)
Chronic kidney disease (CKD) following radical nephrectomy (RN) for renal cell carcinoma (RCC)	Upregulated	renal parenchyma (cortex & medulla) from 71 RCC patients post radical nephrectomy (FFPE) and renal biopsies (cortex) from healthy donors (n = 12)	Not investigated	Hypothesized to modulate TGF-β signaling (linked to TGF-β2 and TGFBR3).	A predictive biomarker for post-operative CKD. Its overexpression is linked to renal inflammation and fibrosis, even in patients with no pre-operative signs of kidney disease	(71)
Alzheimer's Disease (AD)	Upregulated in ABCA1-labeled exosomes	Human serum: Controls (n = 60), SCD (n = 89), MCI (n = 92), DAT (n = 92); CSF; Controls (n = 6), MCI (n = 16), DAT (n = 11)	Human RBCs, Human WBCs, Mouse hippocampal neuron HT-22 cells, Primary mouse neuronal cells	Amyloid precursor protein (APP)	Potential early diagnostic biomarker for AD	(72)

In many disorders, miR-193b levels are lower than normal. This loss of function often leads to dysregulated cell proliferation, inflammation, and tissue damage. For instance, in systemic sclerosis [53] and aortic dissection [54], miR-193b has a role in the regulation of the proliferation and migration of vascular smooth muscle cells, and modulation of vessel wall thickening and instability. Similarly, in “In-Stent Restenosis”, its downregulation leads to neointimal hyperplasia via the target gene PLAU [65].

It is also involved in the pathogenesis of inflammatory and autoimmune conditions. For instance, in psoriasis [60], allergic rhinitis [6] and sepsis [63], low levels of miR-193b result in hyperactivation of inflammatory pathways (like STAT3, NF-κB, and TCR signaling), driving keratinocyte proliferation, inflammatory secretion, and immune system overreaction. Its downregulation is also noted in endometriosis (in the original uterine tissue, potentially enabling cell migration) [69] and in some stages of Parkinson's disease [61], indicating a complex, stage-specific role in neurodegeneration.

Conversely, in several other conditions, elevated miR-193b contributes to disease pathology. For instance, in type 2 diabetes [57] and prediabetes [59], increased miR-193b impairs glucose metabolism by enhancing hepatic glucose production and promoting insulin resistance. In a related finding, it also drives muscle atrophy (sarcopenia) in diabetic patients by disrupting protein synthesis [58].

Additionally, miR-193b contributes to the pathogenesis of neurodegenerative diseases. In Parkinson's disease (particularly late stage) [62] and Alzheimer's disease [72], upregulated miR-193b inhibits neuroprotective pathways (like PGC-1α), leading to mitochondrial dysfunction, oxidative stress, and neuronal damage. It is also investigated as a potential diagnostic biomarker in Alzheimer's disease [72].

In osteoarthritis, its increase with age promotes the degradation of the cartilage matrix [55]. In pulmonary vascular dysfunction linked to diabetes, it is upregulated by metabolic stress and disrupts a critical vasodilatory pathway [68]. It is also upregulated in placenta accreta spectrum [64] and hypertensive disorders of pregnancy [70], where it regulates trophoblast function and is associated with disease severity.

From a mechanistical point of view, miR-193b frequently targets genes to control smooth muscle cell, keratinocyte, and trophoblast growth and movement. In addition, it can be regarded as a key regulator of major pro-inflammatory pathways, including NF-κB, STAT3 [60], and TCR signaling [63]. It is also involved in the glucose metabolism (via YWHAZ/FOXO1 and SOS2) [57], muscle protein balance (via Akt/mTOR) [58], and extracellular matrix remodeling (through regulation of MMPs [54] and ADAMTS5 [55]). Finally, as documented in the context of Parkinson's disease, it regulates mitochondrial function. In fact, its target PGC-1α is a master regulator of mitochondrial biogenesis and antioxidant responses [61].

In addition to the mentioned *in vitro* studies and expression assays in the clinical samples, the significant role of miR-193b in human disorders has been verified in animal models (Table 3). The animal studies described in this table reveal miR-193b as a multifaceted molecule with significant roles in disease pathogenesis. Notably, it can function as a therapeutic target in aortic dissection [54], psoriasis [60], and diabetes-related muscle atrophy [58]. Moreover, miR-193 is a potential responsive biomarker for disease state and intervention efficacy in glucose intolerance [59]. In fact, miR-193 can be suggested as a key node in dysregulated signaling pathways across a spectrum of disorders, particularly in cardiovascular and metabolic diseases.

**Table 3:** Summary of animal studies on the role of miR-193b in non-malignant disorders.

Type of disorder	Animals	Results	References
Aortic Dissection	Apolipoprotein E-deficient (ApoE−/−) male mice (6–8 weeks old)	Silencing H19 (with shH19) or directly increasing miR-193b-3p (with an agomir) in mice model: Reduced physical damage to the aorta (less degeneration, thinner aortic wall). Reversed the abnormal protein expression (increased α-SMA/SM22α; decreased MMP-2/MMP-9).	(54)
Influenza A Virus (IAV) Infection	8-week-old female 57BL/6J mice	Adenovirus-mediated miR-193b delivery tended to reduce body-weight loss and significantly lowered lung viral load in H1N1-infected mice.	(56)
Psoriasis	7-week-old female C57BL/6 mice	Overexpression of miR-193b-3p (agomiR-193b-3p) improved psoriasis symptoms, whereas inhibition (antagomiR-193b-3p) worsened them.	(60)
Myocardial Hypertrophy	TAC-induced mice	Silencing upstream lncRNA N29, which mediates the miR-193b-5p/TGFBR2 axis, improved cardiac function, reduced pathology, and inhibited hypertrophy.	(14)
Exercise-induced pulmonary hypertension (EIPH) in HFpEF	ZSF-1 obese rats (HFpEF model) vs. Lean ZSF-1 rats (controls)	EIPH phenotype: normal resting RVSP, markedly elevated during exercise. Molecular profile: ↑miR-193b, ↓NFYA, ↓sGCβ1, ↓cGMP. Functional impairment: blunted pulmonary arterial vasodilation without significant remodeling.	(68)
Combined pre- and post-capillary PH (CpcPH) in HFpEF	ZSF-1 obese rats treated with SU5416 (CpcPH model) vs. Lean ZSF-1 rats (controls)	CpcPH phenotype: elevated resting RVSP, severe exercise-induced worsening with RV hypertrophy Molecular profile: Same impaired pathway as EIPH model. Structural & functional impairment: Significant PA remodeling and vasodilation deficit. Therapeutic rescue: AAV6-NFYA gene therapy and SGLT2 inhibition both restored sGCβ1-cGMP signaling and improved exercise PH.	(68)
Glucose Intolerance	6-week-old male C57BL/6J mice	Plasma miR-193b was significantly increased in glucose-intolerant HFD-fed mice compared to controls. The exercise intervention that improved glucose tolerance and reduced liver fat significantly decreased circulating miR-193b levels back towards baseline. Mirrored the human findings, validating its value as a responsive biomarker.	(59)
Diabetes and Prediabetes (in a gestational diabetes model)	STZ-treated female mice and their offspring.	miR-193b downregulated in diabetic mothers but upregulated in their offspring; proposed as a biomarker to differentiate diabetic and prediabetic states.	(73)
Alzheimer's Disease (AD)	APP/PS1 double-transgenic mice (C57BL/6J background) vs. matched wild-type	ABCA1-exosomal miR-193b increased in CSF and serum of APP/PS1 mice in an age-dependent manner; exosomes from AD mouse CSF trafficked to serum after injection.	(20)
Type 2 Diabetes (T2D) & Sarcopenia (Muscle Atrophy)	C57BL/6J wild-type and db/db (T2D) mice	miR-193b OE (C57): Induced muscle atrophy, dysfunction, and insulin resistance via PDK1/Akt inhibition. miR-193b KD (db/db): Rescued muscle loss, improved function and insulin sensitivity via PDK1/Akt activation. PDK1 KD (C57): Blocked miR-193b effects, confirming PDK1 as key target.	(58)

## 8 *In silico* identification of miR-193b target genes and pathways

Finally, we assessed miR-193b targets and related signaling pathways using *in silico* tools (Table 4, Table 5). The results strongly suggested that miR-193b is a key regulator of immune response, inflammation, and specific metabolic and signaling processes.

**Table 4:** Kyoto Encyclopedia of Genes and Genomes (KEGG) pathway analysis of top miR-193b target genes.

Term	P-value	Genes
Herpes simplex virus 1 infection	0.004482	ZNF33A; ZNF791; OAS2; CASP3; ZNF506; CCL5; ZNF627; ZNF107; ZNF780B; POU2F2; ZFP28; ZNF431
Cytokine-cytokine receptor interaction	0.00987	CCR1; BMP2; IL23R; CCL5; TNFSF10; TNFRSF11B; TNFRSF1B; CSF2RA
Viral protein interaction with cytokine and cytokine receptor	0.018073	CCR1; CCL5; TNFSF10; TNFRSF1B
Lipid and atherosclerosis	0.021298	CASP3; CCL5; TNFSF10; NFATC2; MAP2K7; POU2F2
TNF signaling pathway	0.026126	CASP3; CCL5; MAP2K7; TNFRSF1B
Influenza A	0.029523	OAS2; CASP3; CCL5; TMPRSS4; TNFSF10
Salmonella infection	0.039495	VPS33A; CASP3; TNFSF10; MAP2K7; RAB7A; ARL8B
Natural killer cell mediated cytotoxicity	0.042683	SHC4; CASP3; TNFSF10; NFATC2
Nicotinate and nicotinamide metabolism	0.047717	NMNAT1; SIRT2
Neomycin, kanamycin and gentamicin biosynthesis	0.049015	GCK

**Table 5:** Gene ontology enrichment analysis of top miR-193b target genes using Enrichr online database (https://maayanlab.cloud/Enrichr/).

GO Term	Index	Name	P-value	Adjusted p-value	Odds Ratio	Combined score
Biological Process	1	Zinc Ion Import Across Plasma Membrane (GO:0071578)	0.002398	0.03458	571.06	3445.29
	2	Protein Branched Polyubiquitination (GO:0141198)	0.005388	0.03458	235.02	1227.64
	3	Intracellular Monoatomic Ion Homeostasis (GO:0006873)	0.005388	0.03458	235.02	1227.64
	4	Regulation of Meiotic Cell Cycle (GO:0051445)	0.005986	0.03458	210.26	1076.21
	5	Anaphase-Promoting Complex-Dependent Catabolic Process (GO:0031145)	0.006284	0.03458	199.74	1012.63
	6	Zinc Ion Transmembrane Transport (GO:0071577)	0.006881	0.03458	181.56	904.00
	7	Bile Acid and Bile Salt Transport (GO:0015721)	0.007477	0.03458	166.42	814.75
	8	Zinc Ion Transport (GO:0006829)	0.007477	0.03458	166.42	814.75
	9	Transition Metal Ion Transport (GO:0000041)	0.009265	0.03721	133.09	623.08
	10	Negative Regulation of Epithelial to Mesenchymal Transition (GO:0010719)	0.01075	0.03721	114.05	516.95
Cellular Component	1	Anaphase-Promoting Complex (GO:0005680)	0.006284	0.03142	199.74	1012.63
Molecular Function	1	Bile Acid:Sodium Symporter Activity (GO:0008508)	0.001499	0.007788	999.50	6499.56
	2	Interleukin-17 Receptor Activity (GO:0030368)	0.002098	0.007788	666.27	4108.59
	3	Solute:Monoatomic Cation Symporter Activity (GO:0015294)	0.002997	0.007788	444.11	2580.41
	4	Monoatomic Cation:Bicarbonate Symporter Activity (GO:0140410)	0.003595	0.007788	363.33	2044.88
	5	Monocarboxylate:Sodium Symporter Activity (GO:0140161)	0.003894	0.007788	333.03	1847.77
	6	Bile Acid Transmembrane Transporter Activity (GO:0015125)	0.005388	0.008974	235.02	1227.64
	7	Bicarbonate Transmembrane Transporter Activity (GO:0015106)	0.006881	0.008974	181.56	904.00
	8	Zinc Ion Transmembrane Transporter Activity (GO:0005385)	0.007179	0.008974	173.66	857.29
	9	Transition Metal Ion Transmembrane Transporter Activity (GO:0046915)	0.01046	0.01162	117.41	535.47
	10	Cytokine Receptor Activity (GO:0004896)	0.02465	0.02465	48.57	179.85

Kyoto Encyclopedia of Genes and Genomes (KEGG) pathway analysis revealed the primary signaling and metabolic pathways that are significantly enriched with predicted miR-193b target genes. The most statistically significant pathways were dominated by viral infections and immune-inflammatory signaling. In details, multiple pathways related to viral pathogens were enriched, including Herpes simplex virus 1 infection, Influenza A, and viral protein interaction with cytokine receptors. Key targeted genes involved in these pathways included ‘CASP3’ (apoptosis), ‘CCL5’ (chemokine), ‘OAS2’ (antiviral response), and ‘TNFSF10’ (TRAIL, involved in immune cell killing). Among immune and inflammatory signaling pathways, “Cytokine-cytokine receptor interaction,” “TNF signaling pathway,” and “Natural killer cell mediated cytotoxicity” were prominently featured. This reinforces a central role for miR-193b in modulating the immune response. Finally, the “Lipid and atherosclerosis” pathway was significantly enriched, linking miR-193b to processes relevant to cardiovascular disease, which aligns with the animal study findings in aortic dissection and pulmonary hypertension.

Gene Ontology analysis categorized the predicted functions of miR-193b target genes into three domains: Biological Process, Cellular Component, and Molecular Function. Among the Biological Process, involvement of miR-193b in processes like “Negative Regulation of T Cell Apoptosis,” “Positive Regulation of T-helper 17 Cell Differentiation,” and “Regulation of T Cell Activation” highlight a specific focus on adaptive immunity. Moreover, miR-193b is implicated in cell differentiation and growth factor signaling and extracellular matrix and transport pointing to potential roles of this miRNA in tissue structure and metabolite transport. “Cellular Component” analysis further indicated involvement of miR-193b in intracellular trafficking and signaling.

Together, these computational predictions paint a coherent picture of miR-193b as a multifaceted regulator whose targets are heavily implicated in the regulation of immune and inflammatory responses, modulation of key signaling pathways, and control of cellular processes, such as apoptosis, maturation, and extracellular matrix dynamics. This predicted role in immune and inflammatory regulation provides a possible molecular explanation for its reported effects in animal models of disorders like psoriasis, viral infection, and cardiovascular disease.

**Fig. 1:**
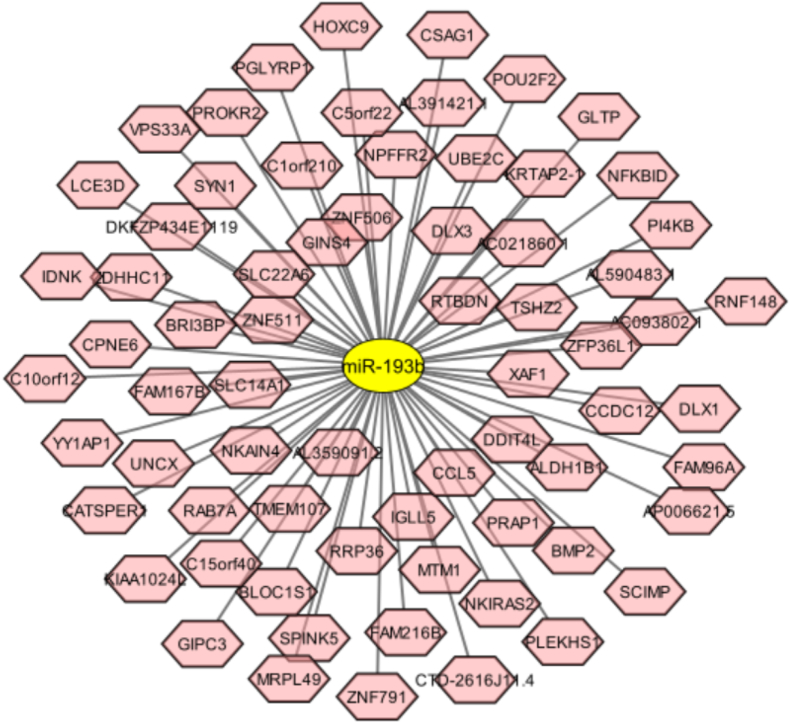
Correlation pairs of miR-193b targets, predicted by TargetScan online tool. The interaction network was constructed by Cytoscape software.

Finally, the predicted miR-193b targets were visualized using the Cytoscape software (Fig. 1).

## 9 Discussion

In the persistent search for novel diagnostic and therapeutic strategies, the field of molecular medicine has turned its focus to miRNAs. miR-193b exemplifies the promise of this class of molecules, functioning as both a critical “brake” on cellular growth and invasion and a promoter of tumorigenesis, based on the related context. Its frequent loss in some types of cancers such as gastric and cervical cancers as well as osteosarcoma positions it not only as a valuable prognostic indicator but also as a potential tool for gene therapy. On the other hand, it has been found to be up-regulated in esophageal and bladder cancers. Meanwhile, data regarding its expression pattern in some types of cancers, such as colorectal, lung and pancreatic cancers is conflicting. Mechanistically, miR-193b exerts its effects by targeting crucial genes and signaling pathways involved in oncogenesis. Differential expression of components of these pathways or ceRNA network interacting with this miRNA in different tissues or different stages of a certain type of cancer might explain the bidirectional or inconsistent expression and function of this miRNA.

Notably, a previous meta-analysis of ten cohort studies has suggested miR-193b as an appropriate biomarker in the cancer prognosis for Asian patients [74], based on the association between down-regulation of miR-193b and poor overall survival rate in diverse human cancers. Furthermore, patients with lower levels of miR-193b have exhibited higher tendency to develop malignancies with higher potential of metastasis [74].

In some populations, to strengthen translational relevance, the diagnostic and prognostic performance of miR-193b across cancers has been reported. For example, in an Iranian cohort, miR-193b with the most area under the curve (AUC: 1.00, 95 % confidence interval 1.00–1.00, P < 0.0001) has exhibited a high discriminatory power for cutaneous melanoma [29]. Also, in another Iranian cohort the ROC curve analyses have confirmed the significance of miR-193b level as a potential biomarker for Parkinson's disease diagnosis (AUC: 0.7925, 95 % confidence interval: 0.6434–0.9416, P = 0.0016) [61]. Overall, across multiple diseases, dysregulated miR-193b expression associates with poor overall survival, supporting additive prognostic stratification alongside conventional clinicopathologic factors.

Beyond oncology, the reach of miR-193b into conditions such as fibrosis and metabolic syndrome suggests a common mechanistic thread in human disease pathogenesis. The data from *in vitro* and in vivo studies suggests that miR-193b has significant clinical potential. It has a potential application as a diagnostic biomarker for prediabetes, Alzheimer's disease, and hypertensive disorders of pregnancy. Moreover, it can be a prognostic/predictive biomarker for assessment of the risk of developing chronic kidney disease after kidney cancer surgery and for the severity of hypertensive disorders of pregnancy.

Finally, miR-193b has emerged as a promising therapeutic candidate because of its dysregulated activity across multiple diseases. Clinical translation requires careful monitoring of pharmacodynamics endpoints, including suppression of target proteins and circulating miRNA changes, alongside rigorous safety evaluation to address off-target effects. Scalable manufacturing of miRNA mimics and standardized preclinical toxicology are essential steps toward bringing miR-193b-based therapies into clinical practice, underscoring its potential as both a biomarker and a therapeutic agent.

In conclusion, miR-193b is a powerful and versatile regulatory miRNA whose dysregulation is a common feature in the pathogenesis of numerous non-malignant diseases. Its pleiotropic nature makes it a compelling subject for further research, both for understanding disease mechanisms and for developing novel diagnostics and therapeutics. This timely update on the functional significance of miR-193b in human disorders and its role in disease-specific signaling networks paves the way for the translational progress towards clinical applications that could restore its protective function in patients.

## Consent of publication

Not applicable.

## Availability of data and materials

The analyzed data sets generated during the study are available from the co-responding author on reasonable request.

## Ethics approval and consent to participate

Not applicable.

## Funding

No funding has been provided for the research.

## CRediT authorship contribution statement

**Sheyda Khalilian:** Conceptualization, Data curation. **Mohadeseh Fathi:** Conceptualization, Data curation. **Sara Arezi:** Data curation. **Soudeh Ghafouri-Fard:** Supervision, Writing – original draft, Writing – review & editing.

## Declaration of competing interest

Authors declare no conflict of interests.

## Data Availability

No data was used for the research described in the article.
