# Supplementary material for: Updates on the role of miR-193b in the pathogenesis of human cancers and other diseases
Source: Biochem Biophys Rep. 2025 Dec 2;45:102392. doi: 10.1016/j.bbrep.2025.102392 (PMC12719069; doi:10.1016/j.bbrep.2025.102392)
Supplement: Multimedia component 1 [file mmc1.docx]

**Supplementary material**

**Data Dictionary for has-miR-193b (miRBase):**

| **Field** | **Entry** |
| --- | --- |
| **Official Name** | hsa‑miR‑193b |
| **miRBase Accession (Precursor)** | MI0003137 |
| **Mature miRNA IDs** | - hsa‑miR‑193b‑3p (**MIMAT0004767**)<br>- hsa‑miR‑193b‑5p (**MIMAT0002819**) |
| **Species Prefix** | hsa = *Homo sapiens* |
| **Genomic Context** | Chromosome 16: 14,303,967–14,304,049 [+ strand] |
| **Precursor Sequence (Stem‑loop)** | guggucucagaauCGGGGUUUUGAGGGCGAGAUGAguuuauguuuuauccAACUGGCCCUCAAAGUCCCGCUuuuggggucau |
| **Mature Sequence (5p)** | AACUGGCCCUCAAAGUCCCGCU |
| **Mature Sequence (3p)** | (from opposite arm; sequence available in miRBase record) |
| **Annotation Confidence** | High (validated in multiple cloning experiments) |
| **Gene Family** | mir‑193 family (RF01895) |

**Glossary/data dictionary**

| **Term** | **Definition** | **Notes** |
| --- | --- | --- |
| **miR‑193a vs. miR‑193b** | Two distinct but related microRNAs encoded by different genomic loci. | miR‑193a is located on chromosome 17q11.2, while miR‑193b is on chromosome 16p13.12. They share sequence similarity but may regulate overlapping as well as distinct targets. |
| **3p vs. 5p** | Refers to the arm of the precursor miRNA hairpin from which the mature miRNA is derived. | **3p** = derived from the 3′ arm; **5p** = derived from the 5′ arm. Both can be functional, but expression levels and biological roles may differ. |
| **Species nomenclature** | Prefixes indicate the organism of origin. | Examples: *hsa‑miR‑193a‑3p* (human), *mmu‑miR‑193b‑5p* (mouse), *rno‑miR‑193a‑3p* (rat). “hsa” = Homo sapiens, “mmu” = Mus musculus, “rno” = Rattus norvegicus. |
